# Nukuhivensiums, Indolo[2,3-*a*]quinoliziniums from the Marquesan Plant *Rauvolfia nukuhivensis*

**DOI:** 10.3390/molecules171012015

**Published:** 2012-10-12

**Authors:** Nicolas J. Martin, Soizic Prado, Gael Lecellier, Olivier P. Thomas, Phila Raharivelomanana

**Affiliations:** 1 EIMS Laboratory UMR 241 EIO, University of French Polynesia, 98702 Faa'a, Tahiti, French Polynesia; Email: Nicolas.martin@upf.pf (N.J.M.); Gael.lecellier@upf.pf (G.L.); 2 MCAM Laboratory UMR 7245 CNRS, National Museum of Natural History, 63 rue Buffon, 75005 Paris, France; Email: Sprado@mnhn.fr; 3 Nice Institute of Chemistry UMR 7272 CNRS-PCRE, University of Nice-Sophia Antipolis, Parc Valrose, 06108 Nice, France

**Keywords:** alkaloid, *Rauvolfia*, antimicrobial, indoles, biosynthesis

## Abstract

The first phytochemical inspection of the Marquesan endemic plant *Rauvolfia nukuhivensis* led to the isolation and structure characterization of two new indolo[2,3‑*a*]quinolizinium derivatives named nukuhivensium (**1**) and *N*_12_-methyl-nukuhivensium (**2**). They feature an aromatic indolo[2,3-*a*]quinolizinium core, substituted at C-2 by a *n*-propyl group, which is unusual in this family of alkaloid derivatives. The structure elucidation was performed on the basis of NMR spectroscopy and especially by interpretation of 2D HMBC correlations. A biosynthetic pathway is proposed on the basis of known enzymatic transformations for this family of natural products. These compounds exhibited low antimicrobial activities.

## 1. Introduction

The highly extended geographic distribution of French Polynesia in the Pacific Ocean has resulted in a relatively high ratio of endemic species spread over the plethora of islands constitutive of this territory, whose distance from the continents may favor the “insular syndrome” leading to more speciation processes [1]. The Marquesas archipelago is one of the most isolated archipelagos of French Polynesia, which have a very original flora within a high endemicity [2,3]. Among them, the plant *Rauvolfia nukuhivensis* (Apocynaceae), locally called “tueiao”, is endemic of the Marquesas archipelago and more precisely of the small island of Nuku Hiva. Although this plant is still used in traditional medicine as a gynecological antiseptic [4], no phytochemical study has been reported so far. Over-exploited because of the frequent use of its bark (macerate) by local communities, the plant is now classified as a critically endangered species, and the description of its chemical constituents is urgently needed before its extinction [5]. Plants of the genus *Rauvolfia* are well known for their prolific biosynthesis of structurally diverse bioactive indole alkaloids, among them ajmaline and its plethora of structural analogues [6]. Nowadays, the biosynthetic genes of these biologically important alkaloids have been identified, which is an important step towards a commercial use of these compounds [7,8,9]. Some phytochemical work has been previously undertaken on Pacific species of the genus *Rauvolfia*. The Scheuer group identified serpentinine, ajmaline, sandwicine, sandwicencine, tetraphylline, tetraphyllicine and mauiensine from the Hawaiian spp. *Rauvolfia sandwicensis* and *R. mauiensis* [10,11]. Later, the Sevenet group identified ajmaline, but not reserpine, in some endemic species of the genus *Rauvolfia* from New Caledonia [12]. 

**Figure 1:**
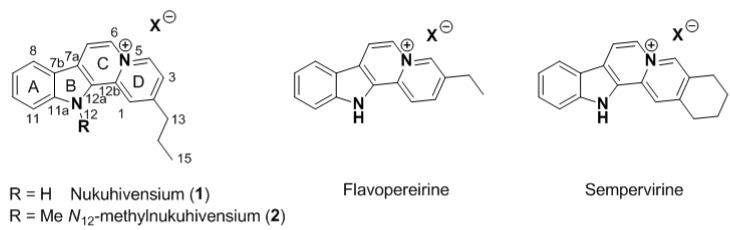
Structures of natural indolo[2,3-*a*]quinoliziniums.

Within the framework of our continued interest in the phytochemical study of endemic and endangered species of French Polynesia, two original indolo[2,3-*a*]quinolizinium derivatives named nukuhivensium (**1**) and *N*_12_-methylnukuhivensium (**2**) were isolated from the Polynesian sp. *Rauvolfia nukuhivensis *(Figure 1). We report herein the isolation and structure elucidation of these new derivatives, as well as their antimicrobial activities.

## 2. Results and Discussion

The stem bark of *Rauvolfia nukuhivensis* was extracted three times with ethanol and the crude extract was submitted to two subsequent fractionation processes, first by reversed phase and then by normal phase chromatography, to yield two main fractions containing alkaloids as revealed by TLC. Purification of one fraction by reversed phase HPLC led to the isolation of both compounds **1** and **2** in their pure form.

The molecular formula of **1** was determined as C_18_H_17_N_2_^+^ by HRESIMS. The ^1^H-NMR data (Table 1) suggested the presence of a polyaromatic system substituted by an *n*-propyl group corresponding to the signals at *δ*_H_ 1.12 (t, ^3^*J* = 7.5 Hz, H_3_-15), 1.94 (tq, ^3^*J* = 7.7 and 7.5 Hz, H_2_-14) and 3.06 (t, ^3^*J* = 7.7 Hz, H_2_-13) ppm which were clearly COSY correlated. Inspection of the chemical shifts of the resulting protons and carbons indicated that the rest of the molecule was polyaromatic. The presence of heterocyclic aromatic systems was deduced from the high deshielding of some resonating protons.

**Table 1:** ^1^H (500 MHz) and ^13^C (125 MHz) NMR data for compounds **1** and **2 **in CD_3_OD.

Position	1			2	
	*δ*_H_, mult (*J* in Hz)	*δ*_C_, mult	HMBC (H→C)	*δ*_H_, mult (*J* in Hz)	*δ*_C_, mult
1	8.65, s	120.5, CH	13, 3, 6, 12a, 12b	8.96, s	121.5, CH
2		154.4, C			154.1, C
3	7.83, d (7.0)	124.4, CH	4, 1, 13	7.86, d (7.0)	123.9, CH
4	9.19, d (7.0)	137.6, CH	3, 6, 2, 12b	9.24, d (7.0)	138.4, CH
6	8.84, d (7.0)	128.1, CH	7, 7a, 12b, 4	8.90, d (7.0)	128.1, CH
7	8.61, d (7.0)	117.2, CH	7b, 6, 12a, 12b	8.66, d (7.0)	116.8, CH
7a		125.0, C			125.6, C
7b		122.5, C			128.9, C
8	8.36, d (8.0)	123.3, CH	11, 7b, 7a	8.41, d (8.0)	122.8, CH
9	7.50, dd (8.0; 7.0)	122.9, CH	11, 8, 10, 11a, 7b	7.54, dd (8.0, 7.0)	123.5, CH
10	7.74, dd (8.0; 7.0)	131.0, CH	8, 7b	7.83, dd (8.0, 7.0)	131.2, CH
11	7.84, d (8.0)	113.6, CH	9, 7b, 11a	7.95, d (8.0)	112.1, CH
11a		143.3, C			144.9, C
12				4.58, s	34.6, CH_3_
12a		131.7, C			131.4, C
12b		134.3, C			135.2, C
13	3.06, t (7.7)	38.6, CH_2_	14, 15, 2, 1, 3	3.09, t (7.7)	38.7, CH_2_
14	1.94, tq (7.7; 7.5)	23.9, CH_2_	15, 13, 2	1.93, tq (7.7, 7.5)	24.4, CH_2_
15	1.12, t (7.5)	14.0, CH_3_	14, 13	1.12, t (7.5)	14.0, CH_3_

**Figure 2:**
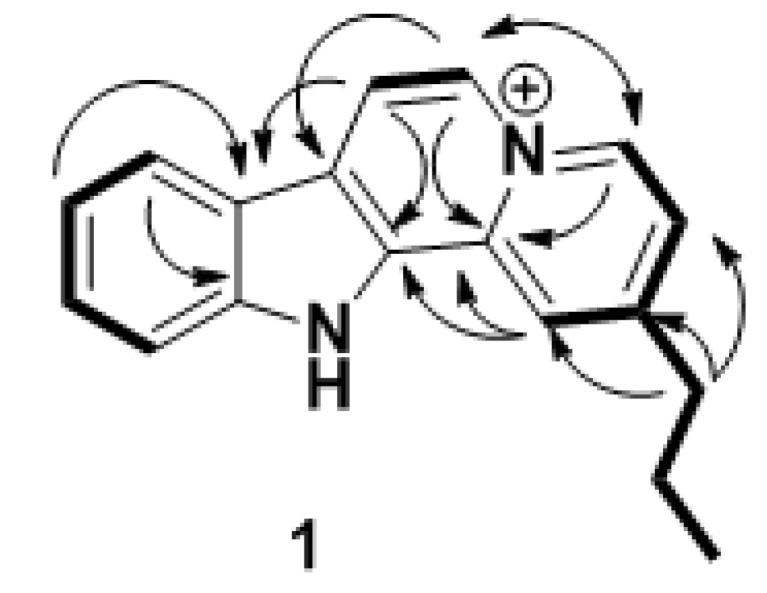
Key COSY (bold) and HMBC (H → C, arrow) correlations for **1**.

A careful inspection of the HMBC spectrum allowed us to unravel the chemical structure of **1**. Indeed, the *n*-propyl was located on the *para* position of a pyridinium ring due to the key H_2_-13/C-2/C-1/C-3 HMBC correlations, the H_2_-13/H-1/H-3 ^4^*J* correlations and the ^3^*J* COSY correlation between the signal at *δ*_H_ 7.83 (d, ^3^*J* = 7.0 Hz, H-3) and 9.19 (d, ^3^*J* = 7.0 Hz, H-4) ppm, this highly deshielded signal being inferred to the vicinity of the electron withdrawing effect of an ammonium group (Figure 2). The clear H-4/C-3/C-2/C-6/C-12b ^2^*J* and ^3^*J* HMBC correlations allowed the assignment of the entire pyridinium D ring as well as the C-6 methine. Starting from this C-6 methine, COSY coupled to the signal at *δ*_H_ 8.61 (d, ^3^*J* = 7.0 Hz, H-7), we were able to build the quinolizinium part of the molecule. Indeed the H-6/C-4/C-12b/C-7/C-7a HMBC correlations yielded the construction of the second pyridinium ring. The pattern of the resulting signals in the ^1^H-NMR and COSY spectra was reminiscent of an indole ring with *δ*_H_ 8.36 (d, ^3^*J* = 8.0 Hz, H-8), 7.50 (dd, ^3^*J* = 8.0 and 7.0 Hz, H-9), 7.74 (dd, ^3^*J* = 8.0 and 7.0 Hz, H-10) and 7.84 (d, ^3^*J* = 8.0 Hz, H-11) ppm. Finally, the indole ring was fused to the second pyridinium at C-12a/C-7a due to the key H-7/C-7b/C-12a/C-7a HMBC correlations. Additional H-8/C-7a/C-11a ^3^*J* HMBC correlations came to confirm the proposed structure of **1**.

The molecular formula of **2** was determined as C_19_H_19_N_2_^+^ by HRESIMS and suggested the presence of an additional methyl group comparing to **1**. This methyl group was easily located at *N*_12_ due to the chemical shifts at *δ*_H_ 4.58 (s, H_3_-C) and *δ*_C_ 33.6 (C-H_3_) ppm (Table 1) which were reminiscent of an aromatic *N*-substituted methyl group. The deshielding of H-1 was clearly induced by the presence of this methyl at *N*_12_ and the H_3_-C/C-11a/C-12a HMBC correlations finally confirmed the substitution at *N*_12_. Compound 2 may be seen as an artifact during our extraction and purification process. Nevertheless, methylation with methanol is quite rare, especially under our mild extraction and purification conditions and the low nucleophilicity of the indole nitrogen.

**Scheme 1:**
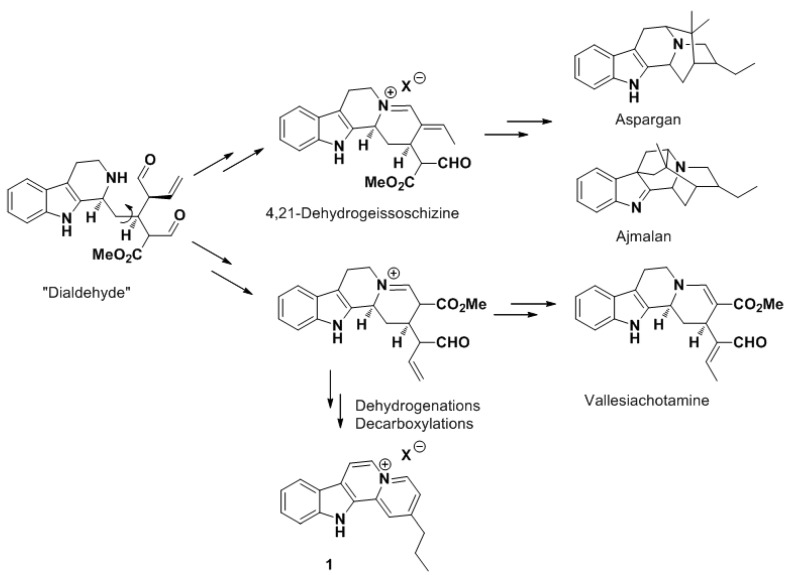
Biosynthetic hypothesis for **1**.

From a structural point of view, these compounds share a rare indolo[2,3-*a*]quinolizinium core already found in flavopereirine [13,14] and sempervirine [15], for example (Figure 1). To our knowledge, the presence of an alkyl chain at C-2 was never reported on this aromatic core and this observation raised the question of the biosynthesis of these compounds. While for flavopereirine and sempervirine an oxidative process starting from saturated known analogues can be proposed, the presence of an *n*-propyl moiety at C-2 was very intriguing. Because there was no doubt on the structure of these compounds, we undertook a detailed analysis of known biochemical pathways leading to these new compounds. We suggest that a plausible hypothesis would start from the key and highly reactive intermediate “dialdehyde” derived from strictosidine (Scheme 1) [16]. In other *Rauvolfia* species, this dialdehyde has been proposed to yield the sarpagan and ajmalan alkaloids via 4,21-dehydro-geissoschizine, which involves an *iso*-propyl at C-2. Rather than an unlikely rearrangement of these three carbon atoms, we propose that nukuhivensiums could be formed like vallesiachotamine (Scheme 1). Indeed, after an alternative cyclization of “dialdehyde” two subsequent decarboxylative steps could afford the *n*-propyl at C-2 and finally compound **1** after additional oxidative steps. Work is ongoing to isolate minor alkaloids from this plant in order to strengthen our biosynthetic hypothesis.

Because extracts of this plant are being used as a gynecological antiseptic, we first decided to assess the antimicrobial potential of these two new derivatives. Compounds **1** and **2** were evaluated *in vitro* for their antimicrobial activity against *Escherichia coli*, *Staphylococcus aureus*, *Candida albicans* and *Aspergillus niger*. Both compounds exhibited low antimicrobial activities against *S. aureus *and *C. albicans *(Table 2). Two options can then be proposed: (i) other minor compounds are responsible of an antimicrobial activty related to the ethnopharmocological use of this plant; or (ii) the mode of action related to the gynecological use of this plant is not as antimicrobial agents.

**Table 2:** Antimicrobial activities of **1** and **2**.

Compounds	MIC_90_ *S. aureus* (µg/mL)	MIC_90_ *E. coli* (µg/mL)	MIC_90_ *C. albicans* (µg/mL)	MIC_90_ *A. niger* (µg/mL)
**1**	105	>150	100	>150
**2**	115	>150	100	>150
Tetracycline	0.5	3	Nt	Nt
Econazole	Nt	Nt	2.60	2.20

Nt: Non tested.

## 3. Experimental

### 3.1. General

UV-Vis spectra were recorded by HPLC-DAD. NMR spectra were measured on a Bruker Avance 500 MHz spectrometer with pulsed field gradient and signals were referenced to the residual solvent signals (CD_3_OD, at *δ*_H_ 3.31 and *δ*_C_ 49.0 ppm). HRESIMS data were measured with a LTQ Orbitrap mass spectrometer (Thermo Finnigan). HPLC purification was carried out on a Waters 600 system equipped with a Waters 717 Plus autosampler, a Waters 998 photodiode array detector, and a Sedex 75 evaporative light-scattering detector (Sedere, France).

### 3.2. Plant Material

*Rauvolfia nukuhivensis* was collected at Maauu in the “Terre Déserte” area on Nuku Hiva Island, Marquesas archipelago, French Polynesia, at 477 m above sea level and identified by Dr Jean-François Butaud. A voucher specimen (JFB 2808) has been deposited in the Herbarium of French Polynesia.

### 3.3. Extraction and Isolation

The dried bark (250 g) was ground and extracted three times with 750 mL of EtOH at room temperature. The maceration was allowed to proceed for 16 h and then the solvent was filtered and concentrated by evaporation to yield a crude oil. The resulting extract (21 g) was dissolved by sonication in a mixture of 200 mL MeOH/CH_2_Cl_2_ (1:1) and then filtered before fractionation. This was carried out by vacuum liquid chromatography on RP-C_18_ and eluted with solvents of decreasing polarity A, B, C, D, E (H_2_O, H_2_O/MeOH (1:1), MeOH, MeOH/CH_2_Cl_2_ (1:1) and CH_2_Cl_2_, respectively). Fraction D, obtained with the MeOH/CH_2_Cl_2_ eluent, provided 2.82 g of an oily residue. A portion of fraction D (1.41 g) was further fractionated by normal phase (diol) flash column chromatography using solvents of stepwise increasing polarity: cyclohexane, cyclohexane/EtOAc (3:1), cyclohexane/EtOAc (1:1), cyclohexane/EtOAc (1:3), EtOAc, EtOAc/MeOH (3:1), EtOAc/MeOH (1:1), EtOAc/MeOH (1:3) and MeOH, yielding nine fractions D1 to D9. Purification of D7 (104 mg) was performed by RP-C_18_ semi-preparative HPLC (Phenomenex, Luna 5 µm C_18_, 250 mm × 10 mm) with a gradient of MeOH/H_2_O/TFA (from 70:30:0.1 to 30:70:0.1, flow 3 mL min^−1^) to afford pure compounds **1** (4.7 mg) and **2** (2.8 mg).

*Nukuhivensium* (**1**). UV measured by HPLC/DAD (MeOH/H_2_O/TFA) *λ*_max_ 224, 241, 290, 340, 382 nm; ^1^H- and ^13^C-NMR see Table 1; HRESIMS *m/z *261.13953 [M]^+^ (calcd for C_18_H_17_N_2_^+^, 261.13917).

N*_12_-methylnukuhivensium* (2). UV measured by HPLC/DAD (MeOH/H_2_O/TFA) *λ*_max_ 225, 237, 248, 287, 335, 393 nm; ^1^H- and ^13^C-NMR see Table 1; HRESIMS *m/z *275.15417 [M]^+^ (calcd for C_19_H_19_N_2_^+^, 275.15482).

### 3.4. Biological Evaluations

Reference strains of *Escherichia coli *(ATCC 8739) and *Staphylococcus aureus* subsp. *aureus* (ATCC 6538) were obtained from the Collection of the Institute Pasteur (Paris, France). Bacterial species were cultivated for 24 h in Luria Bertani medium (LB) at 37 °C. *Candida albicans* (IBMC Strasbourg) and *Aspergillus niger* (ATCC 9142) were cultivated at 30 °C for 48 h in Sabouraud dextrose medium (Sanofi Diagnostic Pasteur). Briefly, pre-cultures of the tested micro-organisms were made by inoculating 10 mL of LB and incubating for 24 h for bacteria or 10 mL in Sabouraud for 48 h for fungi. A culture suspension were made by 1/1,000 dilution from preculture and seeded in 96-well microtitration plates. Four microliters of two-fold serial dilutions of each compound (10 mg/mL) was prepared in 100 μL of medium. The plates were incubated at 37 °C for bacteria and at 30 °C for fungus. After 24 h, the optical density of the bacterial suspension of each well was measured at 595 nm using a multiplate reader. Tetracycline and econazole were used as positive controls.

## 4. Conclusions

Endemic plants of the Pacific Islands have been poorly studied phytochemically and several of them are now recognized as endangered species. Several species of the genus *Rauvolfia* are found on these islands and *Rauvolfia nukuhivensis* is currently used in traditional medicines by the local communities of the Marquesas islands (French Polynesia). Within a program aiming at protecting this species we decided to study the therapeutical potential of its chemodiversity. While ajmaline derivatives are frequently encountered, we were able to isolate two new indolo[2,3-*a*]quinolizinium derivatives closely related to sempervirine, but substituted at C-2 by an *n*-propyl alkyl chain. This finding raised the question of the biosynthesis of these two derivatives and we suggest another type of cyclisation of the well known corresponding dialdehyde. Because these compounds were found to exhibit a low antimicrobial activity, we will follow up the description of the metabolome of this species to further identify the bioactive constituents of this folk medicine.

## Supporting Information

Supplementary materials can be accessed at: http://www.mdpi.com/1420-3049/17/10/12015/s1.
